# Impact of Work-Family Conflict on Sleep Complaints: Results From the Longitudinal Study of Adult Health (ELSA-Brasil)

**DOI:** 10.3389/fpubh.2021.649974

**Published:** 2021-04-21

**Authors:** Aline Silva-Costa, Susanna Toivanen, Lúcia Rotenberg, Maria Carmen Viana, Maria de Jesus M. da Fonseca, Rosane H. Griep

**Affiliations:** ^1^Department of Collective Health, Federal University of Triângulo Mineiro, Uberaba, Brazil; ^2^Department of Public Health Sciences, Stockholm University, Stockholm, Sweden; ^3^National School of Public Health, Oswaldo Cruz Foundation (ENSP/FIOCRUZ), Rio de Janeiro, Brazil; ^4^School of Health, Care and Social Welfare, Mälardalen University, Västerås, Sweden; ^5^Laboratory of Health and Environment Education, Oswaldo Cruz Foundation (IOC/FIOCRUZ), Rio de Janeiro, Brazil; ^6^Department of Social Medicine and Post-graduate Program in Public Health, Federal University of Espírito Santo, Vitória, Brazil

**Keywords:** sleep, work-family conflict, stress, work, epidemiology

## Abstract

**Background:** Balancing work and family demands is often a challenge. Family and job responsibilities may affect many aspects of health, and sleep is an important issue. Work-family conflict (WFC) refers to situations where it is difficult to reconcile family and professional demands. WFC can act in two directions: work-to-family conflicts occur when job demands interfere in family life; family-to-work conflicts arise when family demands interfere with job performance. This study evaluated whether dimensions of WFC—time- and strain-related, work-to-family conflict; family-to-work conflict; and lack of time for self-care and leisure due to work and family demands—were cross-sectionally and longitudinally associated with sleep complaints, by gender.

**Methods:** The sample comprised 9,704 active workers (5,057 women and 4,647 men) from the Brazilian Longitudinal Study of Adult Health (ELSA-Brasil). Standardized questionnaires were used to collect data. WFC was measured at baseline (2008–2010), and sleep complaints were measured at baseline and approximately 4 years after the first visit (2012–2014). To test the association between the four WFC dimensions and sleep complaints, crude and multiple logistic regressions were conducted to estimate odds ratios and 95% confidence intervals. The adjusted model included age, education, marital status, hours worked and work schedule.

**Results:** Mean age at baseline was 48.2 years. Most participants were educated to University degree level (54.5%), married (68.2%) and worked ≤ 40 h/week (66.1%). At baseline, 48.3% of women and 41.1% of men reported sleep complaints. Frequent WFC was reported by women and men, respectively, as follows: time-related work-to-family conflict (32.6 and 26.1%), strain-related work-to-family conflict (25.3 and 16.0%), family-to-work conflict (6.6 and 7.6%) and lack of time for self-care (35.2 and 24.7%). For both women and men, time- and strain-related work-to-family conflicts and conflicts for lack of time for self-care were cross-sectionally and longitudinally associated with sleep complaints. The findings also suggest a weaker and non-significant association between family-to-work conflict and sleep complaints.

**Conclusions:** The statistically significant associations observed here underline the importance of reducing WFC. In the modern world, both WFC and sleep problems are increasingly recognized as frequent problems that often lead to ill health, thus posing a public health challenge.

## Introduction

Work-family conflict (WFC) is defined as an inter-role conflict that arises when the demands and responsibilities of work and family interfere with each other (1, 2). The concept is bidirectional: work-to-family conflict occurs when work demands interfere with family and personal life; family-to-work conflict occurs when family demands interfere with work. WFC can be characterized as time-related (time devoted to work detracts from participation in the family domain and vice versa) and strain-related (the effort required to perform in one domain can impair performance in the other) (2–4). Another form of conflict results when both work and family demands encroach on time available for personal care and leisure (5).

Balancing work and family demands is often a challenge. Family and job responsibilities may affect many aspects of health, where sleep is an important factor (6, 7). Barnes et al. (8) found that sleep may be impaired among those who spend large amounts of time on both work and family activities, given that time is a finite resource. In addition to the availability of time, pressure from efforts to manage life demands may lead to poorer quality of sleep and reduced total sleep time (7).

Spending a great deal of time on work and on the family leaves less time for sleeping (8). Accordingly, as time becomes scarce, time-related WFC can be postulated to have greater impact on sleep duration. On the other hand, the literature on psychosocial job stress and insomnia complaints (9) suggests that strain-related WFC is a stronger influence.

From this perspective, Helsinki City employees who reported strong WFC were more likely to display sleep complaints (10). Both dimensions of WFC (work-to-family and family-to-work conflict) were similarly associated with sleep complaints (10). Work-to-family, but not family-to-work, conflict was associated with both poor sleep quality and shorter sleep in a sample of information technology workers in the United States (11). In Egypt, significant associations were found between high levels of work-to-family, but not family-to-work conflict, and sleep problems (waking up too early and having difficulty falling asleep again or waking up tired after the usual amount of sleep) (12).

Jacobsen et al. (13), from cross-sectional analyses, found higher levels of WFC significantly associated with sleep deficiency, short sleep duration and perceived sleep insufficiency, but not with sleep maintenance problems (13). However, on a longitudinal approach, they did not find WFC to associate significantly with short sleep and sleep maintenance problems (13). Also, they did not consider the directionality of the conflict (13).

Although women's participation in the workforce has increased in recent decades, the division of family tasks between men and women has remained uneven, especially in countries, such as Brazil, with patriarchal social structures (14). The effects of WFC have been observed among both men and women (4, 15, 16), but some studies have reported women experiencing greater WFC (4, 15). The literature has also shown gender and sex dissimilarities in sleep, which may be explained by both physiological and psychological factors (17, 18). Although some studies have pointed to associations between WFC and sleep complaints in diverse countries, to our knowledge, no studies have been conducted in Brazil, where work polices, household responsibility-sharing and gender roles are quite different from those in most developed countries.

This study explored various dimensions of WFC: time, strain, directionality and the interference by work and family demands in time available for personal care and leisure. The study examined whether dimensions of WFC were cross-sectionally and longitudinally associated with sleep complaints, by gender.

## Methods

### Study Design and Participants

The Brazilian Longitudinal Study of Adult Health (ELSA-Brasil) is a prospective cohort study to investigate chronic conditions, such as diabetes and cardiovascular disease. The participants (*n* = 15,105) were recruited (Wave 1: 2008–2010) at five public universities and one research institute in six Brazilian cities. Approximately 4 years after the first visit, 14,014 of these civil servants received a follow-up visit (Wave 2: 2012–2014).

This study included 9,704 active workers (5,057 women and 4,647 men) from that cohort with complete data for all variables. Retirees (*n* = 3,885) and participants with missing data on any variable (*n* = 425) were excluded.

The ELSA-Brasil study was approved by each institution's research ethics committee and Brazil's National Research Ethics Committee. All participants signed written consent forms before data collection.

### Variables of Interest

Standardized questionnaires were used to collect data during in-person visits by standardized, certified teams to the study sites.

#### Work-Family Conflict

In the first wave, WFC was evaluated using a four-item questionnaire validated for Brazilian Portuguese (5). The first three items were based on the model designed by Frone et al. (3, 19), and the fourth item was developed by Pinto et al. (5). The four items included: time-related work-to-family conflict (“work demands keep you from spending the amount of time you would like with your family”), strain-related work-to-family conflict (“work demands make it difficult to fulfill domestic responsibilities, such as caring for the household and children”), family-to-work conflict (“family demands interfere with your responsibilities at work, such as getting to work on time, accomplishing daily tasks, work-related travel and attending meetings outside regular working hours”), and the simultaneous effects of both work and family on lack of time for leisure and self-care (“family and work demands keep you from spending the amount of time you would like on your own care and leisure activities”). For each statement, a five-point, frequency-related response scale was used: never to almost never, rarely, sometimes, often and very often. The categories were grouped into three levels: “never/rarely” (reference category), “sometimes” and “often”(4, 5).

#### Sleep Complaints

Information on sleep complaints was elicited with the following question: “During the past 30 nights, have you had sleep problems (difficulty falling asleep or going back to sleep after waking up in the middle of the night)?” This question derived from the specific section on sleep problems of the Clinical Interview Schedule—Revised (CIS-R) used to assess common mental disorders (20). For the cross-sectional approach, participants who acknowledged having experienced these sleeping problems in Wave 1 were identified as having baseline sleep complaints. For the longitudinal evaluation, those who answered “Yes” in both Waves 1 and 2 were classified in the group with sleep complaints over time; those who answered “No” in both Waves 1 and 2 were classified in the group without sleep complaints over time.

#### Covariates

Data were also collected on age (continuous), sex, education (upper secondary or university/postgraduate), marital status (married/living together or single/living without partner), children under the age of five in the household (Yes/No), hours worked per week (continuous), and work schedule (exclusively day work, former night work and current night work).

### Statistical Analyses

Baseline characteristics were compared, by baseline sleep complaints, using chi-square tests for categorical variables and *t*-tests for continuous variables. To test the association between the four WFC dimensions and sleep complaints (at baseline and over time), crude and multiple logistic regression analyses were conducted to estimate odds ratios (OR) and 95% confidence intervals (CI). The adjusted model included age, education, marital status, hours worked and work schedule. All analyses were performed separately for women and men. Analyses were conducted in R software, version 3.1.2 (R Development Core Team, Vienna, Austria).

## Results

Baseline sample mean age was 48.2 (standard deviation = 6.8) years. Most participants were educated to University level (54.5%), were married (68.2%) and worked ≤ 40 h/week (66.1%). A total of 41.2% of men and 48.3% of women reported sleep complaints at baseline (*p* < 0.001). Frequent WFC was reported as follows: time-related, work-to-family conflict by 32.6% of women and 26.1% of men (*p* < 0.001); strain-related, work-to-family conflict by 25.3 and 16.0%, respectively (*p* < 0.001); family-to-work conflict by 6.6 and 7.6% (*p* = 0.129); and lack of time for self-care by 35.2 and 24.7% (*p* < 0.001).

Table 1 shows descriptions of sociodemographic and work-related characteristics, by baseline sleep complaints. Sleep complaints by both women and men were associated significantly with time- and strain-related work-to-family conflict, and lack of time for self-care.

**Table 1:** Baseline characteristics of the study population according to baseline sleep complaints.

	**Women (*****N*** **=** **5,057)**			**Men (*****N*** **=** **4,647)**		
	**Baseline sleep complaints**			**Baseline sleep complaints**		
	**No**	**Yes**	***p***	**No**	**Yes**	***p***
	**(*n* = 2,615)**	**(*n* = 2,442)**		**(*n* = 2,733)**	**(*n* = 1,914)**	
**Age** (yrs) – Mean [SD]	47.7 [6.8]	48.1[6.4]	0.019	48.5[7.0]	48.4[6.7]	0.787
	*n* (%)	*n* (%)		*n* (%)	*n* (%)	
**Education**						
Upper secondary University	1060 (40.5) 1555 (59.5)	1063 (43.5) 1379 (56.5)	0.033	1314 (48.1) 1419 (51.9)	976 (51.0) 938 (49.0)	0.054
**Marital status**						
Married/living together Non married	1,496 (52.2) 1,119 (42.8)	1,346 (55.1) 1,096 (44.9)	0.142	2,271 (83.1) 462 (16.9)	1,509 (78.8) 405 (21.2)	0.001
**Children**						
5 years old ≤ 5 years old	2,322 (88.8) 293 (11.2)	2,193 (89.8) 249 (10.2)	0.266	2,353 (86.1) 380 (13.9)	1,636 (85.5) 278 (14.5)	0.579
**Work hours**						
≤ 40 h/week >40 h/week	1,860 (71.1) 755 (28.9)	1,719 (70.4) 723 (29.6)	0.587	1,686 (61.7) 1,047 (38.3)	1,153 (60.2) 761 (39.8)	0.333
**Work schedule**						
Day worker Former night worker Current Night worker	2,197 (84.0) 239 (9.1) 179 (6.9)	1,990 (81.5) 265 (10.8) 187 (7.7)	0.054	2,207 (80.8) 347 (12.7) 179 (6.5)	1,503 (78.5) 287 (15.0) 124 (6.5)	0.079
**Time-related work-to-family conflict**						
Never/rarely Sometimes Often	1,073 (41.0) 816 (31.2) 726 (27.8)	876 (35.9) 832 (34.1) 734 (30.0)	0.001	1,195 (43.7) 646 (23.7) 892 (32.6)	726 (37.9) 568 (29.7) 620 (32.4)	<0.001
**Strain-related work-to-family conflict**						
Never/rarely Sometimes Often	1,231 (47.1) 604 (23.1) 780 (29.8)	1,002 (41.0) 677 (27.7) 763 (31.2)	<0.001	1,548 (56.6) 400 (14.6) 785 (28.7)	951 (49.7) 345 (18.0) 618 (32.3)	<0.001
**Family-to-work conflict**						
Never/rarely Sometimes Often	1,802 (68.9) 168 (6.4) 645 (24.7)	1,611 (66.0) 167 (6.8) 664 (27.2)	0.080	1,836 (67.2) 201 (7.3) 696 (25.5)	1,239 (64.7) 153 (8.0) 522 (27.3)	0.221
**Lack of time for self-care**						
Never/rarely Sometimes Often	910 (34.8) 870 (33.3) 835 (31.9)	687 (28.1) 911 (37.3) 844 (34.6)	<0.001	1,270 (46.5) 608 (22.2) 855 (31.3)	730 (38.1) 539 (28.2) 645 (33.7)	<0.001

*Brazilian longitudinal study of adult health (ELSA-Brasil, 2008–2010; N = 9,704)*.

Table 2 shows the results of cross-sectional analyses between WFC and sleep complaints. Higher odds ratios were observed for baseline sleep complaints among women who reported experiencing frequent time- and strain-related work-to-family conflict (OR = 1.22; 95%CI: 1.06; 1.42; and OR = 1.35; 95%CI: 1.17; 1.57, respectively), and frequent lack of time for self-care (OR = 1.37; 95%CI: 1.18; 1.58), than among those who reported experiencing conflict “never/rarely.” For the men, higher odds ratios were observed for baseline sleep complaints among those who reported frequent time- and strain-related work-to-family conflict (OR = 1.50; 95%CI: 1.27; 1.76; and OR = 1.41; 95%CI: 1.18; 1.47, respectively) and frequent lack of time for self-care (OR = 1.56; 95%CI: 1.17; 1.55), than among men who reported experiencing conflict “never/rarely.” In the crude and adjusted analyses, family-to-work conflict was not associated with sleep complaints among either women or men.

**Table 2:** Cross-sectional associations between work-family conflict, lack of time for self-care and sleep complaints at baseline.

	**Baseline sleep complaints**		
	**Crude** ** model** ** OR (95%CI)**	**Adjusted** ** model 1** ** OR (95%CI)**	**Adjusted** ** model 2** ** OR (95%CI)**
**Women (*****n*** **=** **5,057)**			
**Time-related work-to-family conflict**			
Sometimes	1.24 (1.08;1.42)	1.27 (1.10;1.45)	1.23 (1.07;1.42)
Often	1.25 (1.10;1.42)	1.29 (1.12;1.48)	1.22 (1.06;1.42)
**Strain-related work-to-family conflict**			
Sometimes	1.20 (1.06;1.39)	1.24 (1.08;1.41)	1.25 (1.09;1.43)
Often	1.38 (1.20;1.58)	1.43 (1.24;1.65)	1.35 (1.17;1.57)
**Family-to-work conflict**			
Sometimes	1.15 (0.89;1.39)	1.17 (1.03;1.33)	1.12 (0.98;1.28)
Often	1.11 (1.01;1.31)	1.12 (0.89;1.40)	1.05 (0.84;1.32)
**Lack of time for self-care**			
Sometimes	1.34 (1.17;1.54)	1.40 (1.21;1.61)	1.37 (1.19;1.59)
Often	1.39 (1.21;1.59)	1.48 (1.28;1.70)	1.37 (1.18;1.58)
**Men (*****n*** **=** **4,647)**			
**Time-related work-to-family conflict**			
Sometimes	1.14 (1.00;1.31)	1.17 (1.01;1.35)	1.16 (1.01;1.34)
Often	1.45 (1.25;1.67)	1.51 (1.29;1.77)	1.50 (1.27;1.76)
**Strain-related work-to-family conflict**			
Sometimes	1.28 (1.12;1.46)	1.31 (1.14;1.50)	1.28 (1.12;1.47)
Often	1.40 (1.19;1.66)	1.45 (1.22;1.72)	1.41 (1.18;1.47)
**Family-to-work conflict**			
Sometimes	1.11 (0.97;1.27)	1.13 (0.99;1.30)	1.09 (0.95;1.25)
Often	1.13 (0.90;1.41)	1.12 (0.90;1.40)	1.04 (0.83;1.31)
**Lack of time for self-care**			
Sometimes	1.31 (1.14;1.51)	1.36 (1.19;1.57)	1.35 (1.33;1.84)
Often	1.54 (1.33;1.79)	1.66 (1.42;1.95)	1.56 (1.17;1.55)

*Brazilian longitudinal study of adult health (ELSA-Brasil; N = 9,704)*.

*For all models, the reference category is “never/rarely” frequency of work-family conflict at baseline (within each work-family conflict domain). The outcome is baseline sleep complaints: Yes/No. Adjusted model 1: adjusted for age, education, marital status, working hours, and work schedule. Adjusted model 2: adjusted for model 1, plus job strain and depression*.

Sleep complaints at both Waves 1 and 2 were reported by 31.1% (*n* = 1,571) of women and 25.1% (*n* = 1,166) of men. A total of 35.4% (*n* = 1,789) of women and 44.9% (*n* = 2,085) of men did not report sleep complaints over time. Table 3 shows the longitudinal associations between WFC and sleep complaints over time. Among both women and men, sleep complaints over time were associated with work-to-family conflicts and lack of time for self-care, but not with family-to-work conflict. Compared to those who reported experiencing WFC “never/rarely,” the highest odds ratio for sleep complaints over time was observed among men who reported frequent lack of time for self-care (OR = 1.85; 95%CI: 1.51; 2.26).

**Table 3:** Longitudinal associations between work-family conflict, lack of time for self-care and sleep complaints over time.

	**Sleep complaints over time**		
	**Crude** ** model** ** OR (95%CI)**	**Adjusted** ** model 1** ** OR (95%CI)**	**Adjusted** ** model 2** ** OR (95%CI)**
**Women (*****n*** **=** **3,360)**			
**Time-related work-to-family conflict**			
Sometimes	1.40 (1.18;1.66)	1.44 (1.21;1.70)	1.38 (1.16;1.65)
Often	1.47 (1.25;1.72)	1.53 (1.29;1.82)	1.46 (1.22;1.75)
**Strain-related work-to-family conflict**			
Sometimes	1.30 (1.11;1.53)	1.35 (1.15;1.59)	1.37 (1.16;1.62)
Often	1.67 (1.41;1.98)	1.76 (1.47;2.10)	1.65 (1.37;1.98)
**Family-to-work conflict**			
Sometimes	1.23 (1.05;1.44)	1.25 (1.07;1.47)	1.18 (1.00;1.39)
Often	1.26 (0.96;1.67)	1.27 (0.96;1.68)	1.15 (0.86;1.54)
**Lack of time for self-care**			
Sometimes	1.51 (1.27;1.79)	1.58 (1.33;1.88)	1.55 (1.30;1.85)
Often	1.60 (1.35;1.89)	1.71 (1.43;2.03)	1.57 (1.31;1.88)
**Men (*****n*** **=** **3,251)**			
**Time-related work-to-family conflict**			
Sometimes	1.18 (0.99;1.40)	1.20 (1.01;1.43)	1.20 (1.00;1.43)
Often	1.68 (1.40;2.00)	1.73 (1.44;2.10)	1.70 (1.40;2.07)
**Strain-related work-to-family conflict**			
Sometimes	1.42 (1.20;1.67)	1.45 (1.23;1.72)	1.40 (1.18;1.67)
Often	1.76 (1.44;2.14)	1.82 (1.48;2.24)	1.76 (1.42;2.18)
**Family-to-work conflict**			
Sometimes	1.24 (1.06;1.46)	1.26 (1.07;1.49)	1.21 (1.02;1.44)
Often	1.31 (0.99;1.73)	1.31 (0.99;1.73)	1.17 (0.87;1.56)
**Lack of time for self-care**			
Sometimes	1.50 (1.27;1.78)	1.55 (1.31;1.85)	1.52 (1.28;1.81)
Often	1.87 (1.56;2.25)	2.01 (1.66;2.45)	1.85 (1.51;2.26)

*Brazilian Longitudinal Study of Adult Health (ELSA-Brasil; N = 6,611)*.

*For all models, the reference category is “never/rarely” frequency of work-family conflict at baseline (within each work-family conflict domain). The outcome is sleep complaints in waves 1 and 2: Yes/No. Adjusted model 1: adjusted for age, education, marital status, working hours, and work schedule. Adjusted model 2: adjusted for model 1, plus job strain and depression*.

## Discussion

This study of the relationship between WFC and sleep complaints revealed that time- and strain-related, work-to-family conflict, as well as lack of time for self-care, were cross-sectionally and longitudinally associated with sleep complaints among both women and men, regardless of confounding variables. The findings also suggest a weaker and non-significant association between family-to-work conflict and sleep complaints.

The findings for the relationship between WFC and sleep, among both women and men, are consistent with results from previous studies. WFC has been found to be associated with sleep complaints among both women and men, independent of occupational class, marital status, work arrangements and health behavior (10). Nevertheless, contrary to our results, the strength of the association found was greater for women than men (10). Magee et al. (21) found that the association between WFC and poor sleep quality was more marked among men than women.

Given the higher percentages of women often observed experiencing WFC (15, 22) and considering that sleep complains are usually more prevalent among women than men (23, 24), the association between WFC and sleep complaints among men found in this study should be noted. Men still participate much less in domestic and family activities than women. In recent years, however, society has increasingly demanded that men involve themselves in non-work-related tasks. From that perspective, despite traditional gender-related attitudes, men possibly find it difficult to cope with this “new pressure” in the modern world, that is, the need to maintain their performance at work, while having to devote more time to family. Considering work arrangements, Remery and Schippers (22) argued that job flexibility factors (e.g., working hours) can explain the level of WFC perceived by workers. However, these findings are not yet completely understood, and the question remains open.

As with the findings of this study, so other studies found no statistically significant associations between family-to-work conflict and poor sleep (11, 25). Eshak (12) observed, among Egyptians, that both work-to-family and family-to-work conflicts were associated with short sleep duration. On the other hand, he found no significant association between family-to-work conflict and sleep complaints (waking up several times per night, waking up too early and being unable to fall asleep again or waking up tired after a usual amount of sleep) (12). In a sample of information technology industry workers, high levels of work-to-family conflict were associated with poor sleep quality, whereas family-to-work conflict was not (11). In contrast, Lallukka et al. (10) found that both dimensions (work-to-family and family-to-work conflict) were associated with sleep complaints.

Lack of time for self-care has not been explored in depth in the literature and no conclusions can be drawn by comparing with other data. The findings of this study could be explained partly by the link between WFC and unhealthy behavior, such as physical inactivity and unhealthy food habits (26, 27), given that a lifestyle featuring psychosocial stress, unbalanced diet and lacking in physical activity is recognized as a risk factor for sleep problems (28). A recent study of WFC and ideal cardiovascular health scores, using data from ELSA-Brasil, found that workers reporting frequent lack of time for self-care showed lower prevalence of ideal physical activity (29). Also, Svedberg et al. (30), in a study on time pressure, sleep problems and sick leave, argued that the absence of limits between work and leisure can affect sleep and recovery from work (30).

Although these results refer to a pre-pandemic period, recent studies on occupational and behavioral changes relating to the Covid-19 pandemic raised the difficulty of reconciling domestic and family demands, and the related impact on health. The sudden and unexpected changes in workers' routines due to the pandemic have intensified the challenge of defining boundaries between the professional and family spheres. Workers have been forced to create a work area in their homes, and many employees have found themselves working overtime. Also, for those with children at home, it has become harder to focus work tasks. On the other hand, to stop thinking about work is also a challenge, which can increase stress and impair mental health (31). A study conducted during the pandemic showed an association between stress levels and sleep problems that have emerged during the pandemic (32). In that study sample, greater stress was observed in individuals who needed to align with quarantine-related changes in work, those with family responsibilities, those who needed to wake up early and those with chronic illnesses (32).

This study has certain limitations. Firstly, sleep complaints were recorded only twice, with a 4-year interval and with no information on chronic sleep problems. While WFC was measured at the ELSA-Brasil baseline, the questions relating to sleep complaints in the prior month were asked at both baseline and wave 2. In order to investigate the relation between these factors, further information based on more points of measurement can be evaluated over time in future waves of the ELSA-Brasil. Also, recall bias in self-reported sleep patterns could not be ruled out. However, self-reported sleep measurements have been widely used in large epidemiological studies. Given that the ELSA-Brasil participants comprise a sample of specific civil servants, with higher average income and schooling than the national average, the findings should be generalized with caution, since the perception of work-family conflict and its effects on sleep may be influenced by economic status. Despite these limitations, this study assessed WFC carefully, considering its directionality and all dimensions separately, and thus contributes to the literature on WFC and sleep, given the heterogeneity in the studies as to manners of measuring WFC. Although domestic and family responsibilities are still greater for women, this study's findings raise the hypothesis that frequent WFC may also have strong effects on men's sleep, which deserves to be investigated in depth by future studies. Attention is also drawn to the analysis of lack of time for personal care (simultaneous demands by both work and family depriving the individual of time for self-care), which was not explored in previous studies on sleep, and reinforces how detrimental WFC may be to the quality of sleep.

In conclusion, the associations between WFC and sleep patterns underscore the importance of reducing WFC. This constitutes a public health challenge, since both WFC and sleep problems are increasingly recognized as frequent problems in the modern world. WFC reflects the imbalance between work and family, the two most important spheres of adult life. In addition, sleep complaints may also result from the mounting obligations and responsibilities imposed by domestic and work demands. As both WFC and sleep have been investigated as risk factors for a series of chronic diseases, management of these problems may attenuate the adverse effects of WFC and sleep problems, separately or in combination. These findings may contribute to the development and/or improvement of public policies for actions by labor institutions toward work-family balance. In other words, the results offer information that can also be used to raise awareness among managers, workers and their families in building strategies to promote work-family balance. Also, workplace health policymakers need to take a systemic view that regards the human factor as essential to workplace productivity and recognizes that workers' lives are influenced by both family and work. In sum, this is an important and timely issue.

## Data Availability Statement

The ELSA-Brasil study, while open to any researcher, has a policy of requiring that all proposals of investigations pass through the study's publications committee. Requests to access the datasets should be directed to Dr. Rosane H. Griep (rohgriep@gmail.com).

## Ethics Statement

The studies involving human participants were reviewed and approved by the research ethics committees of all six centers (Federal University of Minas Gerais—UFMG: 186/06; São Paulo University—USP: 669/06; Federal University of Rio Grande do Sul—UFRGS: 194/061; Federal University of Espírito Santo—UFES: 041/06; Federal University of Bahia—UFBA: 027/06; Oswaldo Cruz Foundation—FIOCRUZ: 343/06). The patients/participants provided their written informed consent to participate in this study.

## Author Contributions

AS-C wrote the first draft of the manuscript and contributed to data analyses. RG, LR, MF, ST, and MV participated in the study design and provided technical advice during the data analysis. All authors interpreted the results, critically revised the manuscript draft and approved the final version of the manuscript for submission.

## Conflict of Interest

The authors declare that the research was conducted in the absence of any commercial or financial relationships that could be construed as a potential conflict of interest.

## References

[1] Byron K. A meta-analytic review of work-family conflict and its antecedents. J Vocat Behav. (2005) 67:169–198. 10.1016/j.jvb.2004.08.009

[2] Greenhaus JH Beutell NJ. Sources of conflict between work and family roles. Acad Manag Stable. (1985) 10:76–88.

[3] Frone MR Russell M Cooper ML. Relation of work-family conflict to health outcomes: a four-year longitudinal study of employed parents. J Occup Organ Psychol. (1997) 70:325–35. 10.1111/j.2044-8325.1997.tb00652.x

[4] Griep RH Toivanen S van Diepen C Guimarães JMN Camelo LV Juvanhol LL. Work-family conflict and self-rated health: the role of gender and educational level: baseline data from the Brazilian Longitudinal Study of Adult Health (ELSA-Brasil). Int J Behav Med. (2016) 23:372–82. 10.1007/s12529-015-9523-x 26597924 PMC4863030

[5] Pinto KA Menezes GM Griep RH Lima KT Almeida MC Aquino EM. Work-family conflict and time use: psychometric assessment of an instrument in ELSA-Brazil. Rev Saude Publica. (2016) 50:39. 10.1590/S1518-8787.2016050005892 27384968 PMC4926953

[6] Edge CE Cooper AM Coffey M. Barriers and facilitators to extended working lives in Europe: a gender focus. Public Health Rev. (2017) 38:2. 10.1186/s40985-017-0053-8 29450074 PMC5810036

[7] Skinner N Dorrian J. A work-life perspective on sleep and fatigue – looking beyond shift workers. Ind Health. (2015) 53:417–26. 10.2486/indhealth.2015-0009 26027709 PMC4591134

[8] Barnes CM Wagner DT Ghumman S. Borrowing from sleep to pay work and family: expanding time-related conflict to the broader non-work domain. Pers Psychol. (2012) 65:789–819. 10.1111/peps.12002

[9] Yang B Wang Y Cui F Huang T Sheng P Shi T. Association between insomnia and job stress: a meta-analysis. Sleep Breath. (2018) 22:1221–31. 10.1007/s11325-018-1682-y 29959635

[10] Lallukka T Rahkonen O Lahelma E Arber S. Sleep complaints in middle-aged women and men: the contribution of working conditions and work-family conflicts. J Sleep Res. (2010) 19:466–77. 10.1111/j.1365-2869.2010.00821.x 20408929

[11] Buxton OM Lee S Beverly C Berkman LF Moen P Kelly EL. Work-family conflict and employee sleep: evidence from IT workers in the Work, Family and Health Study. Sleep. (2016) 39:1871–82. 10.5665/sleep.6172 27568810 PMC5020369

[12] Eshak ES. Work-to-family conflict rather than family-to-work conflict is more strongly associated with sleep disorders in Upper Egypt. Ind Health. (2019) 57:351–8. 10.2486/indhealth.2018-0091 30101898 PMC6546579

[13] Jacobsen HB Reme SE Sembajwe G Hopcia K Stoddard AM Kenwood C. Work-family conflict, psychological distress, and sleep deficiency among patient care workers. Workplace Health Saf. (2014) 62:282–91. 10.1177/216507991406200703 25000547 PMC4430726

[14] IBGE– Instituto Brasileiro de Geografia e Estatística. Pesquisa Nacional de Amostras por Domicílio – PNAD: Mulheres dedicam mais horas aos afazeres domésticos e cuidado de pessoas, mesmo em situações ocupacionais iguais a dos homens (2018). Available online at: https://agenciadenoticias.ibge.gov.br/agencia-sala-de-imprensa/2013-agencia-de-noticias/releases/24266-mulheres-dedicam-mais-horas-aos-afazeres-domesticos-e-cuidado-de-pessoas-mesmo-em-situacoes-ocupacionais-iguais-a-dos-homens (accessed October 24, 2020).

[15] Leineweber C Baltzer M Magnusson Hanson LL Westerlund H. Work-family conflict and health in Swedish working women and men: a 2-year prospective analysis (the SLOSH study). Eur J Public Health. (2013) 23:710–6. 10.1093/eurpub/cks064 22683777 PMC3719472

[16] Carvalho VS Chambel MJ Neto M Lopes S. Does work-family conflict mediate the associations of job characteristics with employees' mental health among men and women? Front Psychol. (2018) 9:966. 10.3389/fpsyg.2018.00966 29951024 PMC6008497

[17] Mallampalli MP Carter CL. Exploring sex and gender differences in sleep health: a Society for Women's Health Research Report. J Womens Health. (2014) 23:553–62. 10.1089/jwh.2014.4816 24956068 PMC4089020

[18] Suh S Cho N Zhang J. Sex differences in insomnia: from epidemiology and etiology to intervention. Curr Psychiatry Rep. (2018) 20:69. 10.1007/s11920-018-0940-9 30094679

[19] Frone MR Russell M Cooper ML. Antecedents and outcomes of work-family conflict: testing a model of the work-family interface. J Appl Psychol. (1992) 77:65. 10.1037/0021-9010.77.1.65 1556042

[20] Nunes MAA Alves MGM Chor D Schmidt MI Duncan BB. Adaptação transcultural do CIS-R (Clinical Interview Schedule – Revised Version) para o Português no Estudo Longitudinal de Saúde do Adulto (ELSA). Rev HCPA. (2011) 31:487–9.

[21] Magee CA Robinson LD McGregor A. The work-family interface and sleep quality. Behav Sleep Med. (2018) 16:601–10. 10.1080/15402002.2016.1266487 28098478

[22] Remery C Schippers J. Work-family conflict in the european union: the impact of organizational and public facilities. Int J Environ Res Public Health. (2019) 16:4419. 10.3390/ijerph16224419 31718072 PMC6888593

[23] Garland SN Rowe H Repa LM Fowler K Zhou ES Grandner MA. A decade's difference: 10-year change in insomnia symptom prevalence in Canada depends on sociodemographics and health status. Sleep Health. (2018) 4:160–5. 10.1016/j.sleh.2018.01.003 29555129 PMC6203592

[24] Krystal AD Prather AA Ashbrook LH. The assessment and management of insomnia: an update. World Psychiatry. (2019) 18:337–52. 10.1002/wps.20674 31496087 PMC6732697

[25] Crain TL Hammer LB Bodner T Kossek EE Moen P Lilienthal R. Work-family conflict, family-supportive supervisor behaviors (FSSB), and sleep outcomes. J Occup Health Psychol. (2014) 19:155-167. 10.1037/a0036010 24730425 PMC4145734

[26] Roos E Sarlio-Lähteenkorva S Lallukka T Lahelma E. Associations of work-family conflicts with food habits and physical activity. Public Health Nutr. (2007) 10:222–9. 10.1017/S1368980007248487 17288618

[27] Shukri M Jones F Conner M. Relationship between work-family conflict and unhealthy eating: Does eating style matter? Appetite. (2018)123:225–32. 10.1016/j.appet.2017.12.027 29294321

[28] Hafner M Stepanek M Taylor J Troxel WM van Stolk C. Why sleep matters-the economic costs of insufficient sleep: a cross-country comparative analysis. Rand Health Q. (2017) 6:11. 28983434 10.7249/rhq-06-04-11 PMC5627640

[29] Rocco PTP Bensenor IM Griep RH Barreto SM Moreno AB Alencar AP. Work-family conflict and ideal cardiovascular health score in the ELSA-brasil baseline assessment. J Am Heart Assoc. (2019) 8:e012701. 10.1161/JAHA.119.012701 31597505 PMC6818030

[30] Svedberg P Mather L Bergström G Lindfors P Blom V. Time pressure and sleep problems due to thoughts about work as risk factors for future sickness absence. Int Arch Occup Environ Health. (2018) 91:1051–9. 10.1007/s00420-018-1349-9 30128755 PMC6182313

[31] Toniolo-Barrios M Pitt L. Mindfulness and the challenges of working from home in times of crisis. Bus Horiz. (2020) 64:189–97. 10.1016/j.bushor.2020.09.004 33041346 PMC7535863

[32] Robillard R Dion K Pennestri MH Solomonova E Lee E Saad M. Profiles of sleep changes during the COVID-19 pandemic: demographic, behavioural and psychological factors. J Sleep Res. (2021) 30:e13231. 10.1111/jsr.13231 33200477 PMC7744844

